# O *Knockdown* de LncRNA CCAT2 Alivia a Sobrecarga de Pressão ou a Hipertrofia Cardíaca Induzida por Ang II por Meio da Interrupção da Sinalização Wnt/β-catenina

**DOI:** 10.36660/abc.20240181

**Published:** 2024-10-23

**Authors:** Xiaojun Zhang, Zhen Chen, Ning Zhang, Bo Yu, Wei Li, Mengli Zhang, Xian Wu, Ganzhe Liu, Meizhen Dong

**Affiliations:** 1 Qilu Hospital Cheeloo College of Medicine Shangdong University Qingdao Shangdong China Department of Emergency, Qilu Hospital (Qingdao), Cheeloo College of Medicine, Shangdong University, Qingdao, Shangdong – China; 2 The Central Hospital of Wuhan Tongji Medical College Huazhong University of Science and Technology Wuhan Hubei China The Central Hospital of Wuhan, Tongji Medical College, Huazhong University of Science and Technology, Wuhan, Hubei – China

**Keywords:** Cardiomegalia, Cateninas, Angiotensina II

## Abstract

**Fundamento:**

A hipertrofia cardíaca patológica (HC) sustentada é um fator de risco independente para aumento da incidência e mortalidade de eventos cardiovasculares.

**Objetivos:**

Esta pesquisa foi projetada para desvendar o papel do RNA não codificante longo (LncRNA) CCAT2 na progressão da HC.

**Métodos:**

Procedimentos de constrição aórtica transversal (TAC) foram conduzidos para construir um modelo de HC *in vivo* induzido por sobrecarga de pressão. O tratamento com angiotensina II (Ang II) foi utilizado para induzir células hipertróficas de cardiomiócitos de rato H9c2.

**Resultados:**

Os resultados *in vivo* mostraram que o silenciamento de CCAT2 reduziu a área de superfície dos cardiomiócitos, aliviou a fibrose cardíaca e diminuiu os níveis de β-MHC, ANP e BNP em modelos de camundongos HC. Os resultados *in vitro* revelaram que o *knockdown* de CCAT2 reduziu a área de superfície celular e atenuou os níveis de β-MHC, ANP e BNP em células hipertróficas H9c2. Além disso, o silenciamento de CCAT2 diminuiu os níveis de β-catenina ativa, GSK-3β fosforilada e genes alvo Wnt (c-Myc, ciclinaD1 e c-Jun) em camundongos HC e células H9c2 hipertróficas. É importante ressaltar que o tratamento com o ativador da via Wnt / β-catenina LiCl reverteu a supressão do *knockdown* de CCAT2 na área de superfície celular H9c2 e nos níveis de MHC, ANP e BNP.

**Conclusões:**

Coletivamente, o silenciamento do CCAT2 desempenha um papel protetor contra a HC através da inativação da sinalização Wnt/β-catenina, o que sugere que o CCAT2 pode se tornar um alvo terapêutico promissor para o HC.

## Graphical abstract

**Figura Central:**
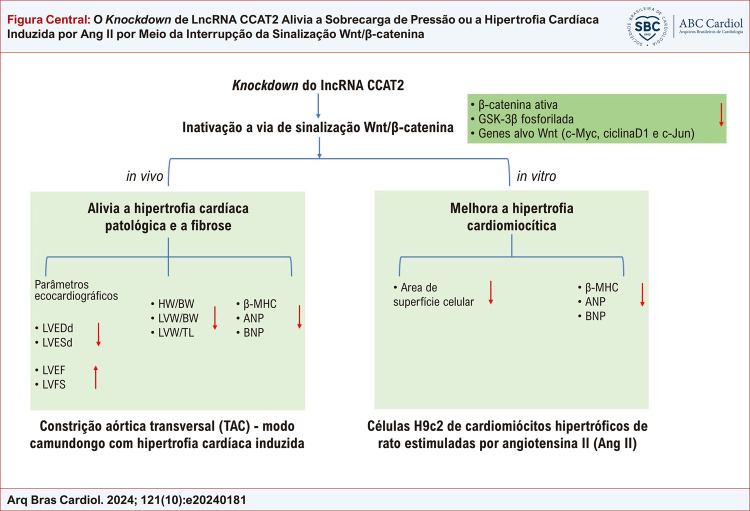
: O *Knockdown* de LncRNA CCAT2 Alivia a Sobrecarga de Pressão ou a Hipertrofia Cardíaca Induzida por Ang II por Meio da Interrupção da Sinalização Wnt/β-catenina

## Introdução

A hipertrofia cardíaca (HC), como reação adaptativa ao estresse hemodinâmico, é responsável pela manutenção da função sistólica cardíaca normal na fase inicial dos estímulos de estresse.^1^ Fatores fisiológicos como exercício e gravidez podem aumentar a carga miocárdica e causar HC, que é considerada um fenômeno normal, sendo leve e reversível.^2^ No entanto, sob a influência de fatores patológicos, como hipertensão e estenose aórtica, se desenvolverá HC patológica, caracterizada por crescimento excessivo do músculo cardíaco, aumento no tamanho e área de superfície das células dos cardiomiócitos, desorganização dos cardiomiócitos e fibrose intersticial do miocárdio.^3^ A HC contínua leva a um aumento no consumo de oxigênio pelo miocárdio, o que causa incompatibilidade entre oferta e demanda do miocárdio e desencadeia ainda vários distúrbios cardiovasculares, incluindo arritmias, insuficiência cardíaca, infarto do miocárdio e morte súbita cardíaca.^4^ Os sintomas comuns de HC incluem dispneia aos esforços, falta de ar, fadiga, aperto no peito, dor no peito, palpitações e síncope.^5^ O sistema renina-angiotensina, responsável pela manutenção da pressão arterial, do equilíbrio hídrico e eletrolítico e da homeostase cardiovascular, consiste em dois eixos principais: o eixo “clássico” que compreende a enzima conversora de angiotensina, a angiotensina II e o receptor tipo 1 da angiotensina (ACE/AngII/AT1) e o eixo “contrarregulador” composto pela enzima conversora de angiotensina 2, angiotensina (1-7) e receptor Mas (ACE2/Ang-(1-7)/Mas).^6^ O eixo clássico exerce efeitos pró-vasoconstritores, pró-inflamatórios, pró-trombóticos e pró-fibróticos, enquanto o eixo contra-regulatório desempenha o papel oposto.^6,7^ Terapias destinadas a inibir a ativação do eixo ECA/AngII/AT1 e/ou promover a função do eixo ECA2/Ang-(1-7)/Mas foram desenvolvidas para o tratamento de doenças cardiovasculares, incluindo HC e insuficiência cardíaca.^8,9^ A fisiopatologia do HC envolve múltiplos sistemas celulares e moleculares, sendo complexa e multifatorial.^10^ Os RNAs não codificantes (ncRNAs) têm atraído atenção considerável nas últimas décadas, com evidências crescentes ligando-os à patogênese do HC e das doenças cardiovasculares.^11^ Portanto, é urgente explorar a etiologia molecular específica da HC para encontrar alvos mais eficazes para o seu tratamento e melhorar a eficácia diagnóstica e terapêutica.

RNAs não codificantes longos (LncRNAs) são transcritos com um comprimento de sequência de mais de 200 nucleotídeos sem potencial de codificação de proteínas.^12^ Um grande conjunto de estudos elucidou que os LncRNAs estão inextricavelmente ligados ao desenvolvimento de doenças humanas através da regulação da expressão gênica em níveis transcricionais e pós-transcricionais.^13^ Até agora, vários LncRNAs foram confirmados como tendo um papel fundamental no desenvolvimento do CH e os mecanismos reguladores subjacentes foram esclarecidos. Por exemplo, o LncRNA ZEB2-AS1 é regulado positivamente em modelos murinos induzidos por constrição aórtica transversa (TAC) de HC e cardiomiócitos primários de camundongos estimulados por fenilefrina, e o silenciamento de ZEB2-AS1 desempenha um papel protetor contra HC através da regulação negativa de PTEN.^14^ O LncRNA NBR2 regulado negativamente em pacientes com HC está negativamente correlacionado com a função cardíaca e o grau da doença, e a superexpressão de NBR2 ativa a via LKB1/AMPK/Sirt1 e enfraquece o estresse do retículo endoplasmático, aliviando assim a hipertrofia induzida por angiotensina II (Ang II) de células miocárdicas humanas.^15^ A expressão de LncRNA MIAT é elevada em células H9c2 derivadas de coração de rato e camundongos submetidos à indução de HC pelo tratamento com Ang II, e o *knockdown* de MIAT mitiga a HC através do aumento do nível de miR-150.^16^ LncRNA CCAT2, localizado no cromossomo 8q24.21, foi originalmente identificado como um oncogene no câncer colorretal.^17^ O locus genômico CCAT2 abriga o SNP rs6983267, que está intimamente relacionado ao aumento do risco de várias doenças malignas. O papel oncogênico do CCAT2 tem sido bem documentado em vários tipos de câncer humano, incluindo câncer de pulmão, mama, gástrico, cervical e de próstata.^18^ No entanto, faltam pesquisas sobre o papel do CCAT2 no HC.

No presente estudo, modelos *in vitro* e *in vivo* de HC foram construídos realizando tratamento com Ang II em células H9c2 de cardiomiócitos de rato e TAC em camundongos, respectivamente. O objetivo da nossa pesquisa é examinar a função do LncRNA CCAT2 na progressão da HC e o mecanismo subjacente.

## Métodos

### Animais

Setenta camundongos C57BL/6 J machos (8 a 10 semanas de idade, 22 a 24 g) foram adquiridos no Jackson Laboratory (Bar Harbor, Maine, EUA). Todos os camundongos foram alojados em condições específicas livres de patógenos, a uma temperatura padrão de 23°C e 55-60% de umidade em um ciclo claro/escuro de 12 horas, com comida e água disponíveis livremente. Os cuidados com os animais e os procedimentos experimentais foram permitidos pelo Comitê Institucional de Cuidados e Uso de Animais do Wuhan Myhalic Biotechnology Co., Ltd sob o número de protocolo HLK-202303112.

### Experimentos animais

**Figura 1:**
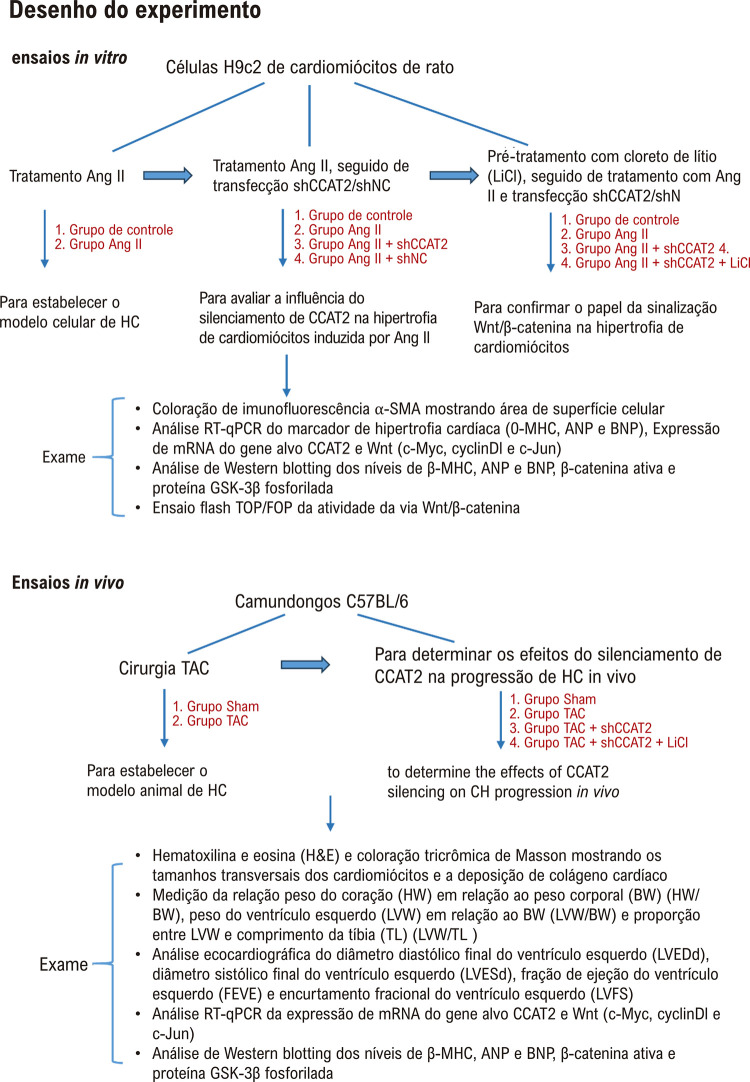
– Desenho do experimento.

**Figura 2:**
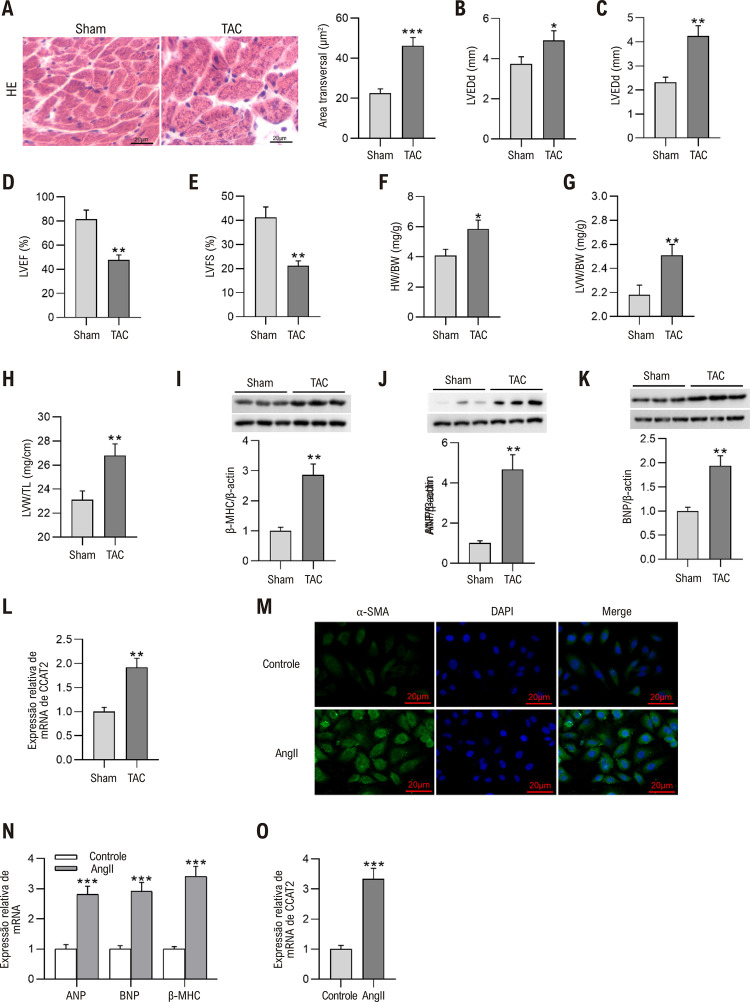
– CCAT2 é regulado positivamente nos tecidos cardíacos de modelos de camundongos HC e células H9c2 hipertróficas tratadas com Ang II. (A) Imagens representativas de coloração H&E de seções cardíacas e quantificação da área transversal de cardiomiócitos em camundongos HC simulados e induzidos por TAC. Barra de escala, 20 μm. n = 6 camundongos por grupo. (B-E) Exame de parâmetros ecocardiográficos, incluindo diâmetro diastólico final do ventrículo esquerdo (LVEDd), diâmetro sistólico final do ventrículo esquerdo (LVESd), fração de ejeção do ventrículo esquerdo (FEVE) e encurtamento fracionário do ventrículo esquerdo (LVFS) de camundongos Sham e induzidos por HC com sistema de ultrassom. n = 6 camundongos por grupo. (F-H) A relação entre o peso do coração (HW) e o peso corporal (BW), o peso do ventrículo esquerdo (LVW) em relação ao BW e a relação entre o LVW e o comprimento tibial (TL) de camundongos HC simulados e induzidos por TAC. n = 6 camundongos por grupo. (I-K) Medição dos níveis de proteína β-MHC, ANP e BNP em tecidos cardíacos de camundongos por Western blotting. n = 6 camundongos por grupo.(L) Detecção da expressão de mRNA de CCAT2 em tecidos cardíacos de camundongos por RT-qPCR. n= 6 camundongos por grupo. (M) Observação do tamanho das células miocárdicas de ratos H9c2 após tratamento com Ang II através da coloração de imunofluorescência α-SMA. Barra de escala, 20 μm. n = 3 repetições. (N) Análise dos níveis de mRNA de β-MHC, ANP e BNP em células H9c2 controle e estimuladas por Ang II por meio de RT-qPCR. n=3 repetições. (O) Avaliação da expressão de mRNA de LncRNA CCAT2 em células H9c2 após estimulação com Ang II. n=3 repetições. Os dados são expressos como médias ± DP. *p < 0,05, **p < 0,01, ***p < 0,001.

A HC induzido por sobrecarga de pressão foi construída por cirurgia TAC conforme descrito anteriormente.^19^ Resumindo, os ratos foram anestesiados com pentobarbital sódico a 0,3% (40 mg/kg; Sigma-Aldrich, St. Louis, MO, EUA) via injeção intraperitoneal após adaptação de uma semana. Em seguida os camundongos receberam toracotomia seguida de dissecção aórtica e constrição transversal da aorta com agulha de calibre 26 e sutura com fio de seda 7-0. Os ratos do grupo Sham receberam a mesma operação sem ligadura transversa da aorta. Após 24 h de operação do TAC, vetores do vírus adeno-associado-9 (AAV9) transportando um shRNA específico para CCAT2 (AAV9-shCCAT2) foram injetados em camundongos modelo HC induzidos por TAC através da veia da cauda para *knockdown* CCAT2, com AAV9-shNC como o controle negativo (Figura 2A). Os ratos foram alocados aleatoriamente em 4 grupos: o grupo Sham (n=10), o grupo TAC (n=20), o grupo TAC + AAV9-shCCAT2 (n=20), e o grupo TAC + AAV9-shNC (n=20). Os camundongos nos grupos TAC + AAV9-shCCAT2 e TAC + AAV9-shNC foram injetados diariamente com 100 μL (1,2×1012 vg/mL) AAV9-shCCAT2 ou AAV9-shNC por 3 semanas, enquanto os ratos nos grupos Sham e TAC foram injetados diariamente com 100 μL de solução salina por 3 semanas. Posteriormente, os ratos foram sacrificados por deslocamento cervical e seus corações foram colhidos para análise posterior. O desenho experimental para os ensaios *in vivo* é apresentado na Figura 1.

### Análise ecocardiográfica

O Vevo 2100 Imaging System (Visual Sonics, Toronto, ON, Canada) foi utilizado para realizar ecocardiografia do ventrículo esquerdo em camundongos 3 semanas após a indução do TAC. Resumidamente, os ratos foram anestesiados por inalação de 1,5% de isoflurano (Sigma-Aldrich), colocado com o peito para cima em uma tábua de exame e limpado para remover todos os pelos do peito. As medidas do modo M das dimensões do ventrículo esquerdo (VE), incluindo o diâmetro sistólico final do VE (LVESd) e o diâmetro diastólico final do VE (LVEDd), foram realizadas usando o cabeçote de varredura e calculadas a média de dez ciclos cardíacos consecutivos. A fração de ejeção (FE) e a fração de encurtamento (FS) do VE foram calculadas com base nos dados de LVESd e LVEDd.

### Avaliação histológica

Os tecidos cardíacos dos camundongos foram fixados em formalina a 10% por 24 h, desidratados em etanol e xileno, embebidos em blocos de parafina e cortados em cortes transversais de 5 μm de espessura. Os cortes foram então corados com hematoxilina e eosina (H&E; LMAI Bio, Xangai, China) e tricrômico de Masson (Solarbio, Pequim, China) para avaliar os tamanhos transversais dos cardiomiócitos e a deposição de colágeno cardíaco, respectivamente. As lâminas foram examinadas microscopicamente, seguido do cálculo da área transversal e da área fibrótica dos cardiomiócitos utilizando o software Image-Pro Plus 6.0 (Media Cybernetics, Silver Spring, MD, USA).

### Tratamento celular e transfecção

Células H9c2 de cardiomiócitos de rato adquiridas da American Type Culture Collection (Manassas VA, EUA) foram cultivados em DMEM (Invitrogen, Carlsbad, CA, EUA) contendo 10% de soro fetal bovino (HyClone, South Logan, UT, EUA) e soluções de penicilina-estreptomicina a 1% (HyClone) e foram mantidas a 37°C em uma incubadora umidificada com 5% de CO_2_. Para estabelecer o modelo *in vitro* de HC, as células H9c2 foram tratadas com Ang II (150 nM; Sigma-Aldrich) por 24 h.^20^ Células H9c2 tratadas com Ang II foram então transfectadas com shCCAT2 ou shNC (RibiBio, Guangzhou, China) usando Lipofectamine 2000 (Invitrogen) para avaliar a influência do silenciamento de CCAT2 na hipertrofia de cardiomiócitos induzida por Ang II. Além disso, para confirmar o papel da via Wnt/β-catenina na hipertrofia de cardiomiócitos mediada por CCAT2, as células H9c2 foram pré-tratadas com cloreto de lítio (LiCl; 2,5 mM; Sigma-Aldrich) por 12 h antes do tratamento com Ang II e transfecção com shCCAT2 ou shNC. O delineamento experimental para os ensaios *in vitro* é apresentado na Figura 1.

### RT-qPCR

O mini kit RNeasy (Qiagen, Valencia, CA, EUA) foi utilizado para extração de RNA total de células H9c2 e tecidos cardíacos de camundongos. Posteriormente, 1 µg de RNA foi purificado usando DNase I (Medchem Express, Monmouth Junction, EUA) e transcrito reversamente em DNA complementar (cDNA) utilizando Superscript II Reverse Transcriptase (Invitrogen). A amplificação e detecção por PCR foram realizadas com o GoTaq qPCR Master Mix (Promega, Madison, WI, EUA) no ABI7500 Sequence Detection System (Applied Biosystems, Foster City, CA, EUA). As sequências iniciadoras estão listadas na Tabela 1. Os níveis de expressão relativa foram calculados com o método 2 ^CT^ após normalização para mRNA de GAPDH.

**Tabela 1:** – Sequências de primers usadas em RT-qPCR

Gene	Espécie	Sequências iniciais
β-MHC	Camundongo	Avanço: 5’-AACCTGTCCAAGTTCCGCAAGGTG-3’
		Reversa: 5'-GAGCTGGGTAGCACAAGAGCTACT-3'
β-MHC	Rato	Avanço: 5’-AAGTGAAGAGCCTCCAGAGTTT-3’
		Reversa: 5'-TGATGAGGCTGGTGTTCTGGG-3'
ANP	Camundongo	Avanço: 5’-CTCCGATAGATCTGCCCTCTTGAA-3’
		Reversa: 5'-GGTACCGGAAGCTGTTGCAGCCTA-3'
ANP	Rato	Avanço: 5’-CCTGGACTGGGGAAGTCAAC-3’
		Reversa: 5'-ATCTATCGGAGGGGTCCCAG-3'
BNP	Camundongo	Avanço: 5’-GCTCTTGAAGGACCAAGGCCTCAC-3’
		Reversa: 5'-GATCCGATCCGGTCTATCTTGTGC-3'
BNP	Rato	Avanço: 5’-TGACGGGCTGAGGTTGTTTT-3’
		Reversa: 5'-ACACTGTGGCAAGTTTGTGC-3'
c-Myc	Camundongo	Avanço: 5’-TCCATCCTATGTTGCGGTCG-3’
		Reversa: 5'-AACCGCTCCACATACAGTCC-3'
c-Myc	Rato	Avanço: 5’-AGCGACACAAGAAGCTTCTG-3’
		Reversa: 5'-CTGAAGCAGCTCCGCCAAAC-3'
ciclinaD1	Camundongo	Avanço: 5’-TCAAGTGTGACCCGGACTG-3’
		Reversa: 5'-ATGTCCACATCTCGCACGTC-3'
ciclinaD1	Rato	Avanço: 5’-TCAAGTGTGACCCGGACTG -3’
		Reversa: 5'-CACTACTTGGTGACTCCCGC-3'
c-Jun	Camundongo	Avanço: 5’-GCACATCACCACTACACCGA-3’
		Reversa: 5'-GGGAAGCGTGTTCTGGCTAT-3'
c-Jun	Rato	Avanço: 5’-GCCACCGAGACCGTAAAGAA-3’
		Reversa: 5'-TAGCACTCGCCCAACTTCAG-3'
GAPDH	Camundongo	Avanço: 5’-TTGTCAAGCTCATTTCCTGGTATG-3’
		Reversa: 5'-GCCATGTAGGCCATGAGGTC-3'
GAPDH	Rato	Avanço: 5’-GACATGCCGCCTGGAGAAAC-3’
		Reversa: 5'-AGCCCAGGATGCCCTTTAGT-3'

### Western blotting

Células H9c2 e tecidos cardíacos de camundongo foram lisados usando *buffer* RIPA contendo coquetel de proteinase (Roche Diagnostics, Mannheim, Alemanha). Em seguida, proteínas iguais foram carregadas em géis de SDS-PAGE a 10% para eletroforese, seguido de transferência para membranas de PVDF (Millipore, Billerica, MA, EUA). As membranas foram incubadas com os anticorpos primários contra β-MHC (SAB2106550, 1/2000, Sigma-Aldrich), ANP (sc-515701, 1/1000, Santa Cruz Biotecnology, Santa Cruz, CA, EUA), BNP (sc-271185, 1/1000, Santa Cruz Biotecnology), β-catenina ativa (ab246504, 1/1000, Abcam,Cambridge, Reino Unido), β-catenina (ab68183, 1/1000, Abcam), GSK-3β fosforilada (ab131097, 1/1000, Abcam), GSK-3β (ab227208, 1/1000, Abcam) e β-actina (ab8227, 1/1000, Abcam) a 4°C durante a noite após ser bloqueadas por 1 h com 5% de leite desnatado. No segundo dia, as membranas foram incubadas com o anticorpo secundário conjugado com HRP (ab205718, 1/2000, Abcam) por 1 h em temperatura ambiente (RT) após serem lavadas com solução TBST (Beyotime, Xangai, China). Os complexos imunes foram visualizados usando o método de quimioluminescência aprimorada (Amersham Pharmacia Biotech, Piscataway, NJ, EUA). O software Image J (NIH, Bethesda, MD, EUA) foi empregado para determinar a densidade óptica de cada proteína. A β-actina foi usada como controle de carga.

### Coloração imunofluorescente

As células H9c2 foram lavadas duas vezes com solução salina tamponada com fosfato (Zeye, Xangai, China), fixadas com paraformaldeído a 4% (Zeye) por 15 min e permeabilizadas com Triton X-100 a 0,2%(Sigma-Aldrich) por 15 minutos. Em seguida, 5% de albumina de soro bovino (Sigma-Aldrich) foi usado para bloquear as células por 30 min em temperatura ambiente, após o que o anticorpo α-SMA (14395-1-AP, 1/1000, Proteintech, Rosemont, IL, EUA) foi adicionado, seguido de incubação durante a noite no escuro a 4 °C.

No dia seguinte, as células passaram por outra incubação de 1 h a 37°C com o anticorpo secundário conjugado com FITC (SA00003-8, 1/100, Proteintech). A coloração nuclear foi obtida com DAPI (Sigma-Aldrich). As lâminas foram observadas em um microscópio de fluorescência axiovert 200 invertido (Carl Zeiss, Gottingen, Alemanha), e a área da superfície celular foi calculada usando o software Image-Pro Plus 6.0.

### Ensaio flash TOP/FOP

Os repórteres de luciferase Topflash ou FOPflash (EMD Millipore, Billerica, MA, EUA) foram usados para detectar a atividade da via de sinalização Wnt. Em resumo, após o tratamento indicado ou transfecção, as células H9c2 foram plaqueadas (5000 células/poço) em placas de 24 poços e privadas de soro durante a noite.

Posteriormente, as células foram transfectadas com 0,2 µg de plasmídeos de expressão TOPflash ou FOPflash utilizando Lipofectamine 2000. As atividades de luciferase TOP/FOP foram medidas utilizando o Luciferase Assay System (Promega,Madison, WI, EUA) 48 h após a transfecção.

### Análise estatística

Todos os dados de pelo menos três experimentos individuais repetidos foram analisados usando o software GraphPad Prism versão 5.0 (GraphPad Software Inc., La Jolla, CA, EUA) e são apresentados como média ± desvio padrão (DP). Comparações duplas e múltiplas foram realizadas com teste-t de Student não pareado e ANOVA unidirecional seguido pelo teste *post hoc* de Tukey. Um valor de p<0,05 foi considerado estatisticamente significativo.

## Resultados

### Taxa de mortalidade de camundongos

Todos os animais toleraram a cirurgia e nenhum camundongo morreu durante a operação. Após três semanas da cirurgia TAC e injeção de AAV, 1 camundongo, 12 camundongos, 11 camundongos e 6 camundongos morreram no grupo Sham, no grupo TAC, no grupo TAC+AAV9-shCCAT2 e no grupo TAC+AAV9-shNC, com taxa de sobrevivência de 90%, 40%, 45% e 70% respectivamente.

### CCAT2 é regulado positivamente nos tecidos cardíacos de modelos de camundongos HC e células H9c2 hipertróficas induzidas por Ang II

Os corações dos ratos foram colhidos para exame histológico 4 semanas após a operação TAC. Como mostrado pelas imagens da coloração H&E, a cirurgia TAC aumentou acentuadamente a área transversal dos cardiomiócitos em camundongos (Figura 2A). A medição ecocardiográfica refletiu que LVEDd e LVESd de corações pós-TAC em 3 semanas foram significativamente maiores, enquanto FEVE e LVFS foram menores do que aqueles de corações Sham (Figura 2B-E), indicando a função cardíaca prejudicada de camundongos operados de TAC. A TAC desencadeou um aumento óbvio na relação peso do coração (PC) em relação ao peso corporal (PC), peso do ventrículo esquerdo (PVE) em relação ao PC e relação entre PVE e comprimento tibial (TL), mostrando uma proporção notavelmente maior de tecido cardíaco em camundongos HC (Figura 2F-H). Além disso, a expressão consideravelmente regulada de marcadores HC (β-MHC, ANP e BNP) foi observada nos tecidos cardíacos de camundongos após a indução de TAC (Figura2I-K). Posteriormente, a expressão de LncRNA CCAT2 em tecidos cardíacos de camundongos após operação TAC foi detectada por RT-qPCR, que revelou que CCAT2 estava superexpresso em tecidos cardíacos hipertróficos vs. tecidos cardíacos normais (Figura 2L), implicando que o LncRNA CCAT2 pode participar no desenvolvimento do HC. Para confirmar ainda mais a função do CCAT2 na HC, um modelo celular foi construído tratando células miocárdicas de rato H9c2 com Ang II. O tamanho das células H9c2 foi avaliado por meio da coloração de imunofluorescência α-SMA, que demonstrou que o tratamento com Ang II demonstrou área celular de cardiomiócitos de ratos (Figura 2M).

Enquanto isso, os cardiomiócitos hipertróficos induzidos por Ang II exibiram expressão significativamente maior de ANP, BNP e β-MHC de mRNA do que os cardiomiócitos de controle (Figura 2N). É importante ressaltar que se descobriu que a expressão de LncRNA CCAT2 em células H9c2 aumentava notavelmente após o tratamento com Ang II (Figura 2O), o que implica ainda o papel potencial do CCAT2 no desenvolvimento do HC.

### Silenciamento de CCAT2 alivia HC e fibrose cardíaca in vivo

**Figura 3:**
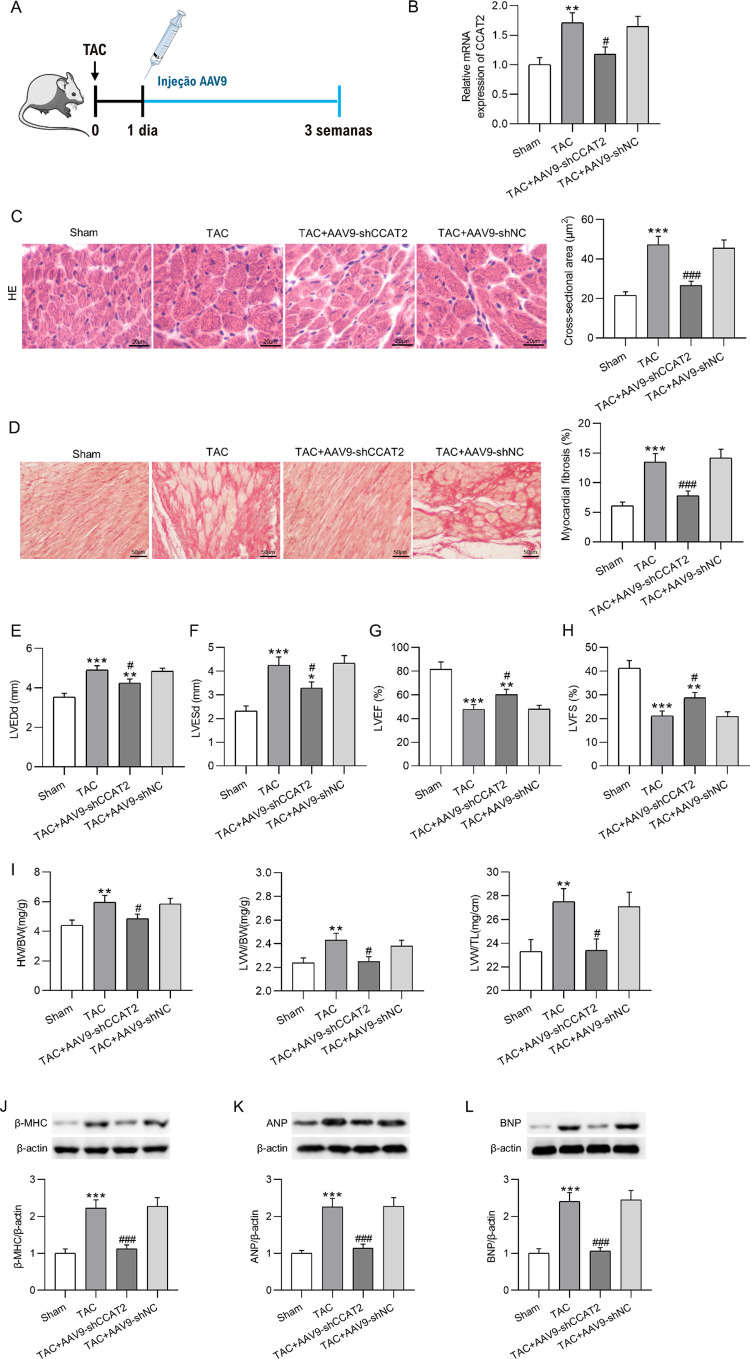
– Silenciamento de CCAT2 alivia HC em ratos modelo. (A) Protocolo para injeção de AAV9-shCCAT2 ou AAV9-shNC em camundongos modelo HC induzidos por TAC. (B) Detecção da expressão de CCAT2 em tecidos cardíacos de camundongos silenciadores de CCAT2 3 semanas após a cirurgia TAC por RT-qPCR. n = 6 camundongos por grupo. (C) Imagens representativas de coloração H&E de seções cardíacas e quantificação da área transversal de cardiomiócitos em camundongos simulados e camundongos HC induzidos por TAC com ou sem knockdown de CCAT2. Barra de escala, 20 μm. n = 6 camundongos por grupo. (D) Imagens representativas de coloração tricrômica de Masson de seções cardíacas e quantificação de fibrose miocárdica. Barra de escala, 50 μm. n = 6 camundongos por grupo. (E-H) LVEDd, LVES, LVEF e LVFS do coração do rato. n = 6 camundongos por grupo.(I) Proporção HW/BW, LVW/BW e LVW/TL de camundongos. n = 6 camundongos por grupo. (J.-L) Determinação dos níveis de proteína β-MHC, ANP e BNP em tecidos cardíacos de camundongos por meio de western blotting. n = 6 camundongos por grupo. Os dados são expressos como médias ± DP. *P < 0,05, **p < 0,01, ***p < 0,001 vs. Sham; #p < 0,05, ###p < 0,001 vs. TAC.

Depois, para determinar o papel detalhado do CCAT2 na regulação do HC *in vivo*, o AAV9-shCCAT2 foi injetado em camundongos modelo HC induzidos por TAC através da veia da cauda para *knockdown* o CCAT2 (Figura 3A). Após três semanas, detectamos e descobrimos que o aumento induzido por TAC na expressão de CCAT2 em tecidos cardíacos de camundongos foi revertido após a depleção de CCAT2 (Figura 3B). A coloração H&E ilustrou que a injeção com AAV9-shCCAT2 reduziu a área de superfície de cardiomiócitos em camundongos HC induzidos por TAC (Figura 3C). Como característica importante do HC patológico, a fibrose cardíaca foi detectada através da coloração tricrômica de Masson. Comparados aos camundongos Sham, os camundongos submetidos à cirurgia TAC apresentaram alterações fibróticas intersticiais e perivasculares nos tecidos cardíacos, acompanhadas de notável acúmulo de colágeno. No entanto, as alterações fibróticas cardíacas induzidas por TAC acima foram melhoradas após o *knockdown* de CCAT2 (Figura 3D). A análise ecocardiográfica mostrou que a elevação induzida por TAC em LVEDd e LVESd e a redução em FEVE e LVFS de corações de camundongos foram marcadamente revertidas após a regulação negativa de CCAT2 (Figura 3 E-H). Além disso, a elevação nas relações HW/BW, LVW/BW e LVW/TL em camundongos causada pela operação TAC foi anulada pelo silenciamento de CCAT2 (Figura 3I). O Western blotting demonstrou que o incremento induzido por TAC nos níveis de proteína β-MHC, ANP e BNP em tecidos cardíacos de camundongos foi antagonizado por injeção com AAV9-shCCAT2 (Figura 3 J-L). No geral, o silenciamento CCAT2 mitiga efetivamente a HC em ratos.

### Silenciamento de CCAT2 melhora a hipertrofia de cardiomiócitos in vitro

**Figura 4:**
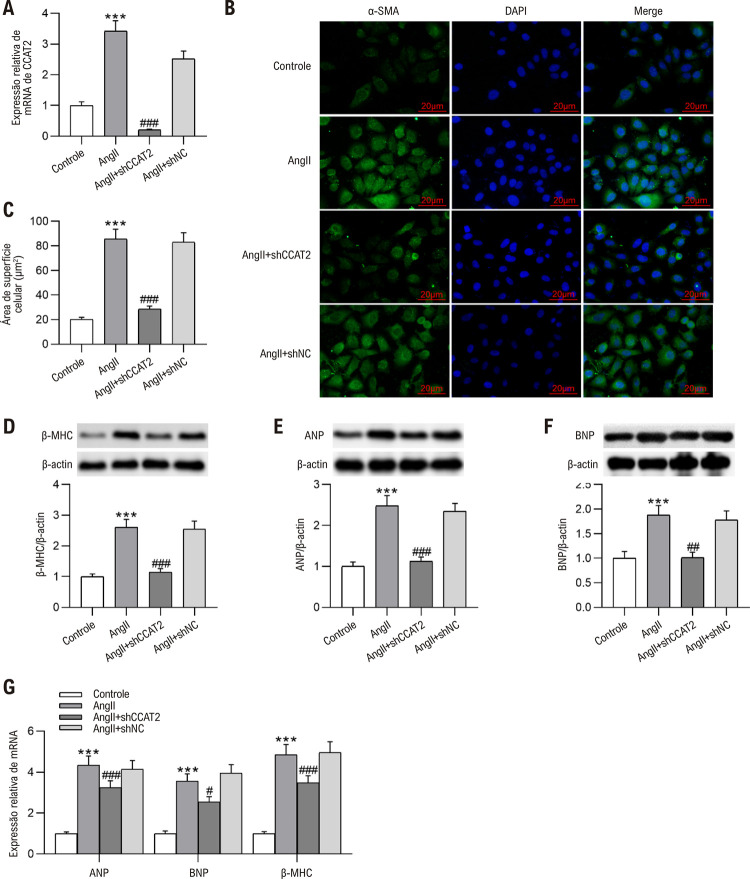
– O silenciamento de CCAT2 melhora a hipertrofia de cardiomiócitos in vitro. Células H9c2 estimuladas com Ang II foram transfectadas com shCCAT2 ou shNC. (A) Análise da expressão de CCAT2 em células H9c2 por RT-qPCR. n=3 repetições. (B-C) Avaliação do tamanho das células H9c2 por coloração imuno-fluorescente com α-SMA. Barra de escala, 20 μm. n=3 repetições. (D-G) Exame dos níveis de β-MHC, ANP e BNP em células H9c2 por western blotting e RT-qPCR. n=3 repetições. Os dados são expressos como médias ± DP. ***P < 0,001 vs Controle; #P < 0,05, ##P < 0,01, ###P < 0,001 vs Ang II.

Da mesma forma, os impactos do *knockdown* de CCAT2 na hipertrofia de cardiomiócitos *in vitro* foram avaliados posteriormente após células H9c2 tratadas com Ang II terem sido transfectadas com shCCAT2. Descobrimos a partir de RT-qPCR que a transfecção shCCAT2 debilitou a promoção do tratamento com Ang II no nível CCAT2 em células H9c2 (Figura 4 A). Conforme revelado pela coloração de imunofluorescência de α-SMA, o *knockdown* de CCAT2 obviamente reduziu a área de superfície em células H9c2 hipertróficas (Figura 4B-C). Além disso, a deficiência de CCAT2 neutralizou o aumento nos níveis de β-MHC, ANP e BNP causados pelo tratamento com Ang II em células H9c2 (Figura 4D-G). Esses dados sugerem que o silenciamento de CCAT2 melhora a hipertrofia de cardiomiócitos *in vitro*.

### O silenciamento do CCAT2 inibe a ativação da sinalização Wnt/β-catenina no CH

**Figura 5:**
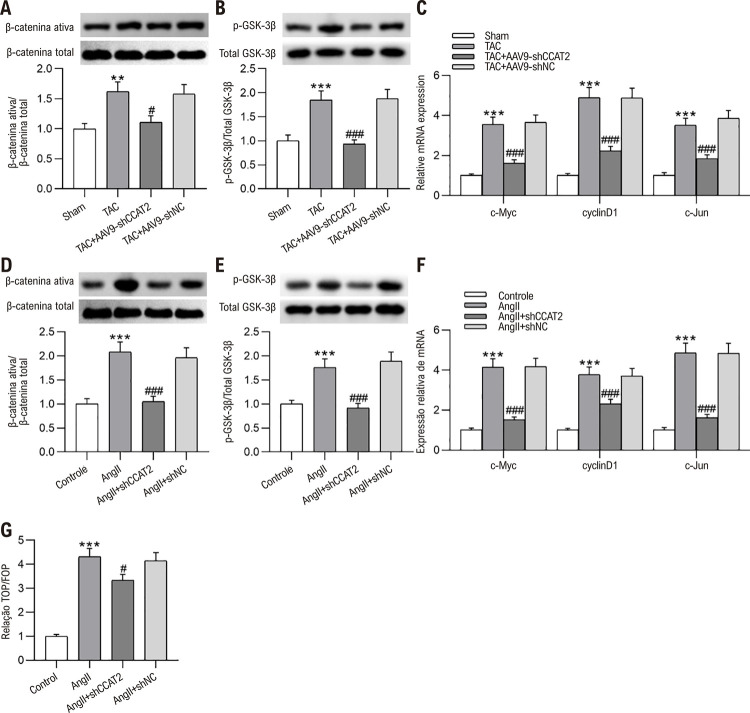
– O silenciamento do CCAT2 inibe a ativação da via Wnt/β-catenina na HC. (A-B) Medição dos níveis de β-catenina ativa e proteína GSK-3β fosforilada em camundongos Sham e camundongos HC induzidos por TAC com ou sem injeção de AAV9-shCCAT2 ou AAV9-shNC por western blotting. n = 6 camundongos por grupo. (C) Detecção de níveis de mRNA de c-Myc, ciclinaD1 e c-Jun em camundongos HC induzidos por TAC após silenciamento de CCAT2 por RT-qPCR. n = 6 camundongos por grupo. Os dados são expressos como médias ± DP. **P < 0,01, ***p < 0,001 vs. Sham; #p < 0,05, ###p < 0,001 vs. TAC. (D-E) Exame dos níveis de β-catenina ativa e proteína GSK-3β fosforilada em células H9c2 de controle e células H9c2 estimuladas por Ang II com ou sem transfecção shCCAT2 ou shNC via western blotting. n= 3 repetições.(F) Avaliação dos níveis de mRNA de c-Myc, ciclinaD1 e c-Jun em células H9c2 tratadas com Ang II após knockdown de CCAT2 por RT-qPCR. n = 3 repetições. (G) Avaliação da atividade da via Wnt/β-catenina em células H9c2 após tratamento com Ang II e transfecção shCCAT2 ou shNC por ensaio flash TOP/FOP. n = 3 repetições. Os dados são expressos como médias ± DP. ***P < 0,001 vs. Controle; #p < 0,05, ###p < 0,001 vs. Ang II.

Para descobrir o mecanismo molecular através do qual o *knockdown* de CCAT2 desempenha um papel protetor contra HC, a influência da depleção de CCAT2 no Wnt/β-cateninaa sinalização foi estimada em modelos de camundongos HC e cardiomiócitos hipertróficos. Conforme mostrado na Figura 5A-B, a cirurgia TAC resultou na ativação da via Wnt/β-catenina em camundongos CH, que, no entanto, foi suprimida pela depleção de CCAT2, conforme evidenciado pela redução nos níveis de β-catenina ativa e proteína GSK-3β fosforilada em camundongos CH silenciados por CCAT2.

A expressão dos genes alvo Wnt foi detectada através de RT-qPCR, que mostrou que a expressão de c-Myc, ciclinaD1 e c-Jun em tecidos cardíacos de camundongos foi marcadamente aumentada após a operação TAC, mas foi atenuada após a injeção de AAV9-shCCAT2 (Figura 5 C). Além disso, a inativação do *knockdown* de CCAT2 na via Wnt/β-catenina no HC foi validada *in vitro*. O incremento induzido por Ang II nos níveis de β-catenina ativa e GSK-3β fosforilada em células H9c2 foi compensado pela regulação negativa de CCAT2 (Figura 5 D-E). RT-qPCR ilustrou que a regulação positiva induzida por Ang II na expressão de c-Myc, ciclinaD1 e c-Jun em células H9c2 foi revertida por *knockdown* de CCAT2 (Figura 5 F). Mais importante ainda, observamos um declínio significativo na relação TOP/FOP em células H9c2 hipertróficas, enquanto o silenciamento de CCAT2 anulou pronunciadamente esse efeito causado por Ang II (Figura 5 G). Em conjunto, o *knockdown* de CCAT2 desempenha um papel inibitório na via Wnt/β-catenina no HC.

### O silenciamento do CCAT2 alivia a hipertrofia dos cardiomiócitos in vitro, restringindo a via Wnt/β-catenina

**Figura 6:**
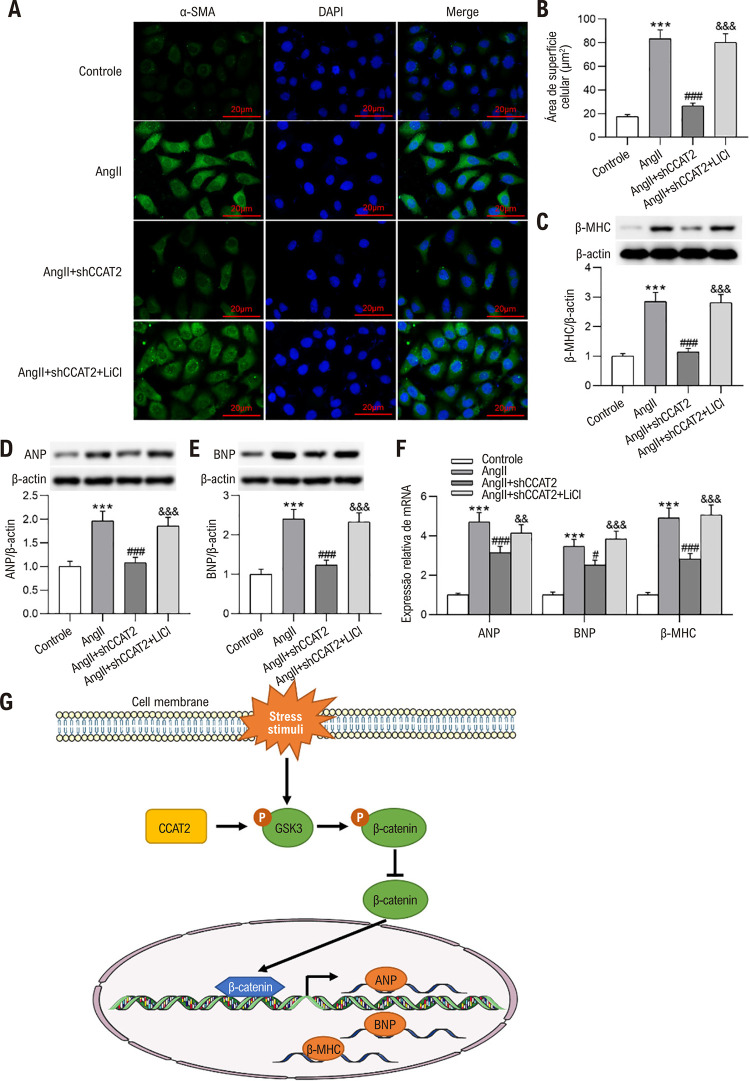
– O silenciamento do CCAT2 alivia a hipertrofia dos cardiomiócitos in vitro ao restringir a via Wnt/β-catenina. Células H9c2 foram pré-tratadas com LiCl antes da estimulação com Ang II e da transfeção com shCCAT2 ou shNC. (A-B) Determinação do tamanho das células H9c2 por coloração imuno-fluorescente com α-SMA. n=3 repetições. Barra de escala, 20 μm. (C-F) Avaliação dos níveis de β-MHC, ANP e BNP em células H9c2 por western blotting e RT-qPCR. n=3 repetições. (G) Diagrama esquemático ilustrando o mecanismo molecular subjacente do LncRNA CCAT2 na hipertrofia cardíaca (CH). Os dados são expressos como médias ± DP. ***P < 0,001 vs Controle; #P < 0,05, ###P < 0,001 vs Ang II; &&P < 0,01, &&&P < 0,001 vs Ang II + shCCAT2.

Para validar se a hipertrofia de cardiomiócitos mediada por CCAT2 é dependente da via Wnt/β-catenina, as células H9c2 foram pré-tratadas com LiCl, um agonista da via Wnt/β-catenina, antes do tratamento com Ang II. De acordo com a coloração de imunofluorescência α-SMA, descobrimos que a inibição do silenciamento de CCAT2 na área de superfície das células H9c2 hipertróficas foi anulada pelo pré-tratamento com LiCl (Figura 6A-B). Conforme ilustrado por western blotting, o pré-tratamento com LiCl reverteu a redução nos níveis de proteína β-MHC, ANP e BNP causada pela depleção de CCAT2 em células hipertróficas H9c2 (Figura 6 C-E). Os resultados do RT-qPCR estavam alinhados com os do western blotting, que demonstraram que o LiCl aboliu o declínio induzido por shCCAT2 nos níveis de mRNA de β-MHC, ANP e BNP em células H9c2 hipertróficas (Figura 6 F). No geral, o LiCl atenua os efeitos preventivos do silenciamento do CCAT2 na hipertrofia dos cardiomiócitos induzida por Ang II.

## Discussão

Este estudo fornece evidências que apoiam os efeitos de alívio da regulação negativa do LncRNA CCAT2 na HC. Foi demonstrado que o silenciamento de CCAT2 atenua a hipertrofia de cardiomiócitos induzida por Ang II *in vitro* e melhora a hipertrofia miocárdica induzida por sobrecarga de pressão em modelos de camundongos. Mecanicamente, o *knockdown* de CCAT2 inativou a sinalização Wnt/β-catenina, e a suplementação de LiCl, um agonista da sinalização Wnt/β-catenina, diminuiu o efeito anti-hipertrófico do silenciamento de CCAT2. As evidências acima indicam que o silenciamento do CCAT2 exerce um efeito anti-hipertrófico no CH, que depende da interrupção da sinalização Wnt/β-catenina.

O acúmulo de evidências provou que os LncRNAs exercem efeitos promotores ou inibitórios na progressão do HC através de uma diversidade de mecanismos. Por exemplo, Wen et al. sugeriram que o PEG10 foi regulado positivamente na HC patológica, e o PEG10 agravou a HC através do aumento da expressão de HOXA9.^21^ Zhou et al. descobriram que o UCA1 era altamente expresso nos cardiomiócitos hipertróficos construídos e nos modelos murinos da HC, e o UCA1 facilitou a progressão do HC aumentando a expressão de HOXA9 através da ligação competitiva com o miR-184.^22^ Em contrapartida, outros estudos indicaram os efeitos protetores de alguns LncRNAs no desenvolvimento da HC. Song et al. divulgaram que a expressão de ADAMTS9-AS1 foi reduzida em pacientes com cardiomiopatia hipertrófica obstrutiva e células de cardiomiócitos humanos tratados com isoproterenol, e a superexpressão de ADAMTS9-AS1 reprimiu a hipertrofia de cardiomiócitos regulando positivamente o KAT7 através da esponja miR-185-5p.^23^ Wang et al. relataram que tanto os modelos celulares quanto os animais de HC apresentaram expressão diminuída de H19, e a superexpressão de H19 prejudicou o desenvolvimento de HC através da modulação do eixo miR-145-3p/SMAD4.^24^ Anteriormente, o papel detalhado e o mecanismo molecular do LncRNA CCAT2 em doenças humanas, especialmente o câncer, foram elucidados. Por exemplo, a deficiência de CCAT2 restringe o crescimento celular e a metástase, mas induz a parada G0/G1 e a apoptose no câncer de mama através da inativação da via de sinalização TGF-β.^25^ CCAT2 atenua a radiossensibilidade e a apoptose de células cancerígenas do esôfago regulando negativamente o eixo miR-145/p70S6K1 e a via de sinalização p53.^26^ CCAT2 promove o crescimento e a metástase de células cancerígenas gástricas por meio da modulação do splicing alternativo do CD44 por meio da ligação ao ESRP1.^27^ No entanto, não foi explorado se o CCAT2 exerce efeitos regulatórios na HC. Aqui, descobrimos que o nível de LncRNA CCAT2 era consideravelmente maior em modelos de camundongos HC induzidos por TAC e células H9c2 tratadas com Ang II. Além disso, o silenciamento de CCAT2 aliviou substancialmente a HC em camundongos e células hipertróficas H9c2, o que foi validado pela inibição da expressão dos principais marcadores HC (β-MHC, ANP e BNP). Isto sugere o efeito potenciador do LncRNA CCAT2 na progressão da HC.

A via canônica de sinalização Wnt/β-catenina é bem conhecida por seu papel regulador no desenvolvimento embrionário, na homeostase de células-tronco e na regeneração de tecidos.^28^ Nos últimos anos, evidências acumuladas demonstraram que a ativação anormal desta via no coração está subjacente à fisiopatologia da hipertrofia miocárdica e da lesão miocárdica.^29^ Foi confirmado que a β-catenina participa do metabolismo dos cardiomiócitos, afetando assim a função e o desenvolvimento do coração.^30^ Estudos provaram que a superexpressão de β-catenina pode resultar em HC.^31,32^ A superexpressão de β-catenina em miócitos cardíacos aumenta o volume celular, induz a formação de actina, ativa marcadores de hipertrofia patológica e eleva os níveis de ANP e BNP.^33^ A HC pode ser melhorada por meio de *knockdown* ou inibição da β-catenina miocárdica.^34^ Zhao et al. relataram que múltiplos ligantes Wnt foram induzidos e a β-catenina foi ativada em ratos com infusão crônica de Ang II e cardiomiócitos primários de ratos, e o bloqueio da via Wnt / β-catenina pelo ICG-001 atenuou a HC e a fibrose miocárdica, bem como inibiu a expressão gênica do marcador hipertrófico.^35^ Portanto, a supressão da sinalização Wnt/β-catenina pode ser considerada um alvo terapêutico promissor para o HC. Recentemente, muitos estudos revelaram que o CCAT2 participa na mediação do desenvolvimento do câncer através da modulação da sinalização Wnt/βcatenina. Por exemplo, o *knockdown* de CCAT2 restringe o crescimento celular e a metástase e facilita a parada do ciclo celular e a apoptose no câncer de tireoide, atenuando a atividade da cascata Wnt/β-catenina.^36^ Os efeitos inibitórios sobre o crescimento celular do câncer de próstata, ciclo celular, transição epitelial-mesenquimal e metástase induzidos pelo silenciamento de CCAT2 foram antagonizados pelo tratamento com o ativador da via Wnt/β-catenina LiCl.^37^ A regulação negativa do CCAT2 impede comportamentos biológicos malignos das células do carcinoma espinocelular oral através da inativação da sinalização Wnt / β-catenina.^38^ Com base na literatura acima, especulamos que o CCAT2 também poderia mediar o desenvolvimento da HC modulando a sinalização Wnt/β-catenina. Aqui, observamos que a regulação positiva nos níveis de β-catenina ativa e genes-alvo GSK-3β fosforilados e Wnt c-Myc, ciclinaD1 e c-Jun induzidos por TAC em camundongos HC e por Ang II em cardiomiócitos hipertróficos foi anulada silenciando o CCAT2. É importante ressaltar que o tratamento com LiCl anulou a supressão do *knockdown* de CCAT2 na área de superfície celular e nos níveis de β-MHC, ANP e BNP em células H9c2 hipertróficas, sugerindo que o silenciamento de CCAT2 dificulta a progressão de HC ao inativar a sinalização Wnt / β-catenina.

Para ser honesto, existem algumas limitações em nosso estudo. Primeiro, ao medir o tamanho dos cardiomiócitos de camundongos, o coração deve estar em diástole, portanto a área dos cardiomiócitos que obtivemos pode ser menor que o tamanho real. Em segundo lugar, para elucidar ainda mais o papel do CCAT2 na patogênese da HC, são necessários diferentes silenciamentos condicionais específicos do tipo de célula de camundongos CCAT2 para investigações adicionais. Terceiro, a expressão de CCAT2 em tecidos de insuficiência cardíaca humana permanece incerta. Amostras de tecidos de pacientes com insuficiência cardíaca terminal devem ser coletadas e analisadas em estudos futuros. Finalmente, mais estudos são necessários para explorar o mecanismo subjacente detalhado através do qual o CCAT2 inativa a via de sinalização Wnt/β-catenina durante a HC.

Até o momento, este é o primeiro relatório a demonstrar que o silenciamento de CCAT2 tem um efeito inibitório na hipertrofia de cardiomiócitos induzida por Ang II in vitro e HC induzida por TAC in vivo, reprimindo a sinalização Wnt/β-catenina (Figura 6G). CCAT2 tem o potencial de fornecer um alvo para o desenvolvimento de terapêuticas clínicas mais eficazes para HC.

Células H9c2 estimuladas com Ang II foram transfectadas com shCCAT2 ou shNC. (A) Análise da expressão de CCAT2 em células H9c2 através de RT-qPCR. n = 3 repetições. (B-C) Avaliação do tamanho das células H9c2 por imunofluorescência de α-SMA. Barra de escala, 20 μm. n = 3 repetições. (D-G) Exame dos níveis de β-MHC, ANP e BNP em células H9c2 por western blotting e RT-qPCR. n= 3 repetições. Os dados são expressos como médias ± DP. ***P < 0,001 vs. Controle; ^#^p < 0,05, ^##^p < 0,01, ^###^p < 0,001 vs. Ang II.

As células H9c2 foram pré-tratadas com LiCl antes da estimulação com Ang II e transfecção shCCAT2 ou shNC. (A-B) Determinação do tamanho da célula H9c2 por meio da coloração de imunofluorescência α-SMA. n = 3 repetições. Barra de escala, 20 μm. (C-F) Avaliação dos níveis de β-MHC, ANP e BNP em células H9c2 através de western blotting e RT-qPCR. n = 3 repetições.(G) Diagrama esquemático que ilustra o mecanismo molecular subjacente do LncRNA CCAT2 em HC. Os dados são expressos como médias ± DP.***p < 0,001 vs. Controle; ^#^p < 0,05, ^###^p < 0,001 vs. Ang II; ^&&^p < 0,01, ^&&&^p < 0,001 vs. Ang II + shCCAT2.
