# Supplementary material for: O Knockdown de LncRNA CCAT2 Alivia a Sobrecarga de Pressão ou a Hipertrofia Cardíaca Induzida por Ang II por Meio da Interrupção da Sinalização Wnt/β-catenina
Source: Arq Bras Cardiol. 2024 Oct 23;121(10):e20240181. [Article in Portuguese] doi: 10.36660/abc.20240181 (PMC11634213; doi:10.36660/abc.20240181)
Supplement: Translation

# Translation: LncRNA CCAT2 Knockdown Alleviates Pressure Overload or Ang II-Induced Cardiac Hypertrophy Via Disruption of the Wnt/β-Catenin Signaling

**DOI:** 10.36660/abc.20240181i

**Authors:** Xiaojun Zhang, Zhen Chen, Ning Zhang, Bo Yu, Wei Li, Mengli Zhang, Xian Wu, Ganzhe Liu, Meizhen Dong

**Affiliations:** 1 China Department of Emergency, Qilu Hospital (Qingdao), Cheeloo College of Medicine, Shangdong University, Qingdao, Shangdong – China; 2 China The Central Hospital of Wuhan, Tongji Medical College, Huazhong University of Science and Technology, Wuhan, Hubei – China

**Keywords:** Cardiomegaly, Catenins, Angiotensin II

## Abstract

**Background:**

Sustained pathological cardiac hypertrophy (CH) is an independent risk factor for increased incidence and mortality of cardiovascular events.

**Objectives:**

This research was designed to unravel the role of long non-coding RNA (LncRNA) CCAT2 in CH progression.

**Methods:**

Transverse aortic constriction (TAC) procedures were conducted to construct a pressure overload-induced in vivo CH model. Angiotensin II (Ang II) treatment was utilized to induce hypertrophic rat cardiomyocyte H9c2 cells.

**Results:**

In vivo results showed that silencing of CCAT2 reduced cardiomyocyte surface area, alleviated cardiac fibrosis, and decreased β-MHC, ANP, and BNP levels in CH mouse models. In vitro results revealed that CCAT2 knockdown reduced cell surface area and attenuated β-MHC, ANP, and BNP levels in hypertrophic H9c2 cells. Besides, CCAT2 silencing decreased the levels of active β-catenin, phosphorylated-GSK-3β, and Wnt target genes (c-Myc, cyclinD1, and c-Jun) in CH mice and hypertrophic H9c2 cells. Importantly, treatment with the Wnt/β-catenin pathway activator LiCl reversed the suppression of CCAT2 knockdown on H9c2 cell surface area and MHC, ANP, and BNP levels.

**Conclusions:**

Collectively, CCAT2 silencing plays a protective role against CH through inactivating the Wnt/β-catenin signaling, which suggests that CCAT2 might become a promising therapeutic target for CH.

## Introduction

Cardiac hypertrophy (CH), as an adaptive reaction to hemodynamic stress, is responsible for maintaining normal cardiac systolic function at the early stage of stress stimuli.^1^ Physiological factors such as exercise and pregnancy may increase the myocardial load and cause CH, which is considered a normal phenomenon, being mild and reversible.^2^ However, under the influence of pathological factors such as hypertension and aortic stenosis, pathological CH will develop, which is characterized by cardiac muscle overgrowth, an increase in cardiomyocyte cell size and surface area, cardiomyocyte disorganization, and myocardial interstitial fibrosis.^3^ Continuous CH leads to an increase in myocardial oxygen consumption, which causes myocardial supply-demand mismatch and further triggers multiple cardiovascular disorders, including arrhythmias, heart failure, myocardial infarction, and sudden cardiac death.^4^ Common symptoms of CH include exertional dyspnea, shortness of breath, fatigue, chest tightness, chest pain, palpitations, and syncope.^5^ The renin-angiotensin system, which is responsible for the maintenance of blood pressure, water and electrolyte balance, and cardiovascular homeostasis, consists of two major axes: the “classical” axis comprising angiotensin-converting enzyme, angiotensin II, and angiotensin type 1 receptor (ACE/AngII/AT1) and the “counter-regulatory” axis comprising angiotensin-converting enzyme 2, angiotensin (1-7), and Mas receptor (ACE2/Ang- (1-7)/Mas).^6^ The classical axis exerts pro-vasoconstrictive, pro-inflammatory, pro-thrombotic, and pro-fibrotic effects, while the counter-regulatory axis plays the opposite role.^6,7^ Therapies aimed at inhibiting the activation of the ACE/AngII/AT1 axis and/or promoting the function of the ACE2/Ang-(1-7)/Mas axis have been developed for the treatment of cardiovascular diseases, including CH and heart failure.^8,9^ The pathophysiology of CH involves multiple cellular and molecular systems, being complex and multifactorial.^10^ Non-coding RNAs (ncRNAs) have attracted considerable attention in recent decades, with increasing evidence linking them to the pathogenesis of CH and cardiovascular disease.^11^ Therefore, it is under great urgency to explore the specific molecular etiology of CH to find more effective targets for its treatment and improve diagnostic and therapeutic efficacies.

Long non-coding RNAs (LncRNAs) are transcripts with a sequence length of more than 200 nucleotides without protein-coding potential.^12^ A large body of studies have elucidated that LncRNAs are inextricably linked to human disease development through gene expression regulation at transcriptional and post-transcriptional levels.^13^ Till now, multiple LncRNAs have been confirmed to play a pivotal role in CH development and the underlying regulatory mechanisms have been clarified. For example, LncRNA ZEB2-AS1 is upregulated in both transverse aortic constriction (TAC)-induced murine models of CH and phenylephrine-stimulated primary mouse cardiomyocytes, and silencing of ZEB2-AS1 plays a protective role against CH through downregulating PTEN.^14^ Downregulated LncRNA NBR2 in patients with CH is negatively correlated with cardiac function and disease degree, and overexpressing NBR2 activates the LKB1/AMPK/Sirt1 pathway and weakens endoplasmic reticulum stress, thereby alleviating angiotensin II (Ang II)-induced hypertrophy of human myocardial cells.^15^ LncRNA MIAT expression is elevated in rat heart-derived H9c2 cells and mice subjected to induction of CH by Ang II treatment, and MIAT knockdown mitigates CH through increasing miR-150 level.^16^ LncRNA CCAT2, which is located on chromosome 8q24.21, was originally identified as an oncogene in colorectal cancer.^17^ The CCAT2 genomic locus harbors the SNP rs6983267, which is closely related to increased risks for various malignancies. The oncogenic role of CCAT2 has been well-documented in various human cancers, including lung, breast, gastric, cervical, and prostate cancers.^18^ Nevertheless, there lack of research on the role of CCAT2 in CH.

In the present study, *in vitro* and *in vivo* models of CH were constructed by performing Ang II treatment in rat cardiomyocyte H9c2 cells and TAC in mice, respectively. The purpose of our research is to scrutinize the function of LncRNA CCAT2 in CH progression and the underlying mechanism.

## Methods

### Animals

Seventy male C57BL/6 J mice (8–10 weeks old, 22–24 g) were purchased from the Jackson Laboratory (Bar Harbor, Maine, USA). All mice were housed under specific pathogen-free conditions at a standard temperature of 23°C and 55–60% humidity on a 12-h light/dark cycle with food and water freely available. The animal care and experimental procedures were permitted by the Institutional Animal Care and Use Committee of the Wuhan Myhalic Biotechnology Co., Ltd under the protocol number HLK-202303112..

### Animal experiments

The pressure overload-induced CH was constructed by TAC surgery as described previously.^19^ In short, mice were anesthetized with 0.3% pentobarbital sodium (40 mg/kg; Sigma-Aldrich, St. Louis, MO, USA) via intraperitoneal injection after one-week adaptation. Then, mice received thoracotomy, followed by aortic dissection and transverse aorta constriction using a 26-gauge needle and a 7-0 silk thread suture. Mice in the Sham group received the same operation without transverse aortic ligation. After 24 h of TAC operation, adeno-associated virus-9 (AAV9) vectors carrying a CCAT2-specific shRNA (AAV9-shCCAT2) were injected into TAC-induced CH model mice through the tail vein to knockdown CCAT2, with AAV9-shNC as the negative control (Figure 2A). Mice were randomly allocated into 4 groups: the Sham group (n=10), the TAC group (n=20), the TAC + AAV9-shCCAT2 group (n=20), and the TAC + AAV9-shNC group (n=20). Mice in the TAC + AAV9-shCCAT2 and the TAC + AAV9-shNC groups were daily injected with 100 μL (1.2×10^12^ vg/mL) AAV9-shCCAT2 or AAV9-shNC for 3 weeks, while mice in the Sham and TAC groups were daily injected with 100 μL saline for 3 weeks. Afterward, mice were sacrificed by cervical dislocation, and their hearts were harvested for further analysis. The experimental design for the *in vivo* assays is presented in Figure 1.

**Figure 2:** – CCAT2 is upregulated in the cardiac tissues of CH mouse models and Ang II-treated hypertrophic H9c2 cells. (A) Representative H&E staining images of heart sections and quantification of cardiomyocyte cross-sectional area in sham and TAC-induced CH mice. Scale bar, 20 μm. n=6 mice per group. (B-E) Examination of echocardiographic parameters including left ventricle end-diastolic diameter (LVEDd), left ventricle end-systolic diameter (LVESd), left ventricular ejection fraction (LVEF), and left ventricle fractional shortening (LVFS) of sham and TAC-induced CH mice with an ultrasound system. n=6 mice per group. (F-H) The heart weight (HW) to body weight (BW) ratio, left ventricle weight (LVW) to BW ratio, and LVW to tibial length (TL) ratio of sham and TAC-induced CH mice. n=6 mice per group. (I-K) Measurement of β-MHC, ANP, and BNP protein levels in mouse cardiac tissues by western blotting. n=6 mice per group. (L) Detection of CCAT2 mRNA expression in mouse cardiac tissues by RT-qPCR. n=6 mice per group. (M) Observation of the size of rat myocardial cells H9c2 after Ang II treatment through α-SMA immunofluorescence staining. Scale bar, 20 μm. n = 3 repetitions. (N) Analysis of β-MHC, ANP, and BNP mRNA levels in control and Ang II-stimulated H9c2 cells through RT-qPCR. n=3 repetitions. (O) Assessment of LncRNA CCAT2 mRNA expression in H9c2 cells upon Ang II stimulation. n=3 repetitions. Data are expressed as the means ± SD. *P < 0.05, **p < 0.01, ***p < 0.001.

**Figure 1:** – The experimental design.

### Echocardiographic analysis

The Vevo 2100 Imaging System (Visual Sonics, Toronto, ON, Canada) was used to perform echocardiography of the left ventricle in mice 3 weeks after TAC induction. Briefly, mice were anesthetized by inhalation of 1.5% isoflurane (Sigma-Aldrich), placed chest up on an examination board, and wiped clean to remove all the chest hair. The M-mode measurements of left ventricular (LV) dimensions including LV end-systolic diameter (LVESd) and LV end-diastolic diameter (LVEDd) were conducted using the scan head and averaged from ten consecutive cardiac cycles. The LV ejection fraction (EF) and fractional shortening (FS) were calculated based on LVESd and LVEDd data.

### Histological assessment

The heart tissues of mice were fixed in 10% formalin for 24 h, dehydrated in ethanol and xylene series, embedded into paraffin blocks, and sliced into 5-μm-thick cross-sectional sections. The sections were then stained with hematoxylin and eosin (H&E; LMAI Bio, Shanghai, China) and Masson’s trichrome (Solarbio, Beijing, China) to evaluate the cross-sectional sizes of cardiomyocytes and the cardiac collagen deposition, respectively. The slides were examined microscopically, followed by the calculation of the cross-sectional area and the fibrotic area of cardiomyocytes using Image-Pro Plus 6.0 software (Media Cybernetics, Silver Spring, MD, USA).

### Cell treatment and transfection

Rat cardiomyocyte H9c2 cells acquired from American Type Culture Collection (Manassas VA, USA) were cultivated in DMEM (Invitrogen, Carlsbad, CA, USA) containing 10% fetal bovine serum (HyClone, South Logan, UT, USA) and 1% penicillin-streptomycin solutions (HyClone) and were maintained at 37°C in a humidified 5% CO_2_ incubator. To establish the *in vitro* model of CH, H9c2 cells were treated with Ang II (150 nM; Sigma-Aldrich) for 24 h.^20^ Ang II-treated H9c2 cells were then transfected with shCCAT2 or shNC (RibiBio, Guangzhou, China) using Lipofectamine 2000 (Invitrogen) to assess the influence of CCAT2 silencing on Ang II-induced cardiomyocyte hypertrophy. Besides, to confirm the role of the Wnt/β-catenin pathway in CCAT2-mediated cardiomyocyte hypertrophy, H9c2 cells were pretreated with lithium chloride (LiCl; 2.5 mM; Sigma-Aldrich) for 12 h before Ang II treatment and shCCAT2 or shNC transfection. The experimental design for the *in vitro* assays is presented in Figure 1.

### RT-qPCR

The RNeasy mini kit (Qiagen, Valencia, CA, USA) was used for total RNA extraction from H9c2 cells and mouse heart tissues. Subsequently, 1 µg RNA was purified using DNase I (Medchem Express, Monmouth Junction, USA) and reverse transcribed to complementary DNA (cDNA) utilizing Superscript II Reverse Transcriptase (Invitrogen). PCR Amplification and detection were conducted with the GoTaq qPCR Master Mix (Promega, Madison, WI, USA) on the ABI7500 Sequence Detection System (Applied Biosystems, Foster City, CA, USA). Primer sequences are listed in Table 1. The relative expression levels were calculated with the 2^CT^ method after normalization to GAPDH mRNA.

**Table 1:** – Primer sequences used in RT-qPCR

Gene	Species	Primer sequences
β-MHC	Mouse	Forward: 5’-AACCTGTCCAAGTTCCGCAAGGTG-3’
		Reverse: 5’-GAGCTGGGTAGCACAAGAGCTACT-3’
β-MHC	Rat	Forward: 5’-AAGTGAAGAGCCTCCAGAGTTT-3’
		Reverse: 5’-TGATGAGGCTGGTGTTCTGGG-3’
ANP	Mouse	Forward: 5’-CTCCGATAGATCTGCCCTCTTGAA-3’
		Reverse: 5’-GGTACCGGAAGCTGTTGCAGCCTA-3’
ANP	Rat	Forward: 5’-CCTGGACTGGGGAAGTCAAC-3’
		Reverse: 5’-ATCTATCGGAGGGGTCCCAG-3’
BNP	Mouse	Forward: 5’-GCTCTTGAAGGACCAAGGCCTCAC-3’
		Reverse: 5’-GATCCGATCCGGTCTATCTTGTGC-3’
BNP	Rat	Forward: 5’-TGACGGGCTGAGGTTGTTTT-3’
		Reverse: 5’-ACACTGTGGCAAGTTTGTGC-3’
c-Myc	Mouse	Forward: 5’-TCCATCCTATGTTGCGGTCG-3’
		Reverse: 5’-AACCGCTCCACATACAGTCC-3’
c-Myc	Rat	Forward: 5’-AGCGACACAAGAAGCTTCTG-3’
		Reverse: 5’-CTGAAGCAGCTCCGCCAAAC-3’
cyclinD1	Mouse	Forward: 5’-TCAAGTGTGACCCGGACTG-3’
		Reverse: 5’-ATGTCCACATCTCGCACGTC-3’
cyclinD1	Rat	Forward: 5’-TCAAGTGTGACCCGGACTG -3’
		Reverse: 5’-CACTACTTGGTGACTCCCGC-3’
c-Jun	Mouse	Forward: 5’-GCACATCACCACTACACCGA-3’
		Reverse: 5’-GGGAAGCGTGTTCTGGCTAT-3’
c-Jun	Rat	Forward: 5’-GCCACCGAGACCGTAAAGAA-3’
		Reverse: 5’-TAGCACTCGCCCAACTTCAG-3’
GAPDH	Mouse	Forward: 5’-TTGTCAAGCTCATTTCCTGGTATG-3’
		Reverse: 5’-GCCATGTAGGCCATGAGGTC-3’
GAPDH	Rat	Forward: 5’-GACATGCCGCCTGGAGAAAC-3’
		Reverse: 5’-AGCCCAGGATGCCCTTTAGT-3’

### Western blotting

H9c2 cells and mouse heart tissues were lysed using RIPA buffer containing proteinase cocktail (Roche Diagnostics, Mannheim, Germany). Then, equal proteins were loaded onto 10% SDS-PAGE gels for electrophoresis, followed by transferring them to PVDF membranes (Millipore, Billerica, MA, USA). The membranes were incubated with the primary antibodies against β-MHC (SAB2106550, 1/2000, Sigma-Aldrich), ANP (sc-515701, 1/1000, Santa Cruz Biotechnology, Santa Cruz, CA, USA), BNP (sc-271185, 1/1000, Santa Cruz Biotechnology), active β-catenin (ab246504, 1/1000, Abcam, Cambridge, UK), β-catenin (ab68183, 1/1000, Abcam), phosphorylated-GSK-3β (ab131097, 1/1000, Abcam), GSK-3β (ab227208, 1/1000, Abcam), and β-actin (ab8227, 1/1000, Abcam) at 4°C overnight following being blocked for 1 h with 5% non-fat milk. On the second day, the membranes were incubated with the HRP-conjugated secondary antibody (ab205718, 1/2000, Abcam) for 1 h at room temperature (RT) after being washed with TBST solution (Beyotime, Shanghai, China). The immune complexes were visualized by using the enhanced chemiluminescence method (Amersham Pharmacia Biotech, Piscataway, NJ, USA). Image J software (NIH, Bethesda, MD, USA) was employed to determine the optical density of each protein. β-actin was used as the loading control.

### Immunofluorescent staining

H9c2 cells were washed twice with phosphate-buffered saline (Zeye, Shanghai, China), fixed with 4% paraformaldehyde (Zeye) for 15 min, and permeabilized with 0.2% Triton X-100 (Sigma-Aldrich) for 15 min. Then, 5% bovine serum album (Sigma-Aldrich) was used to block cells for 30 min at RT, after which α-SMA antibody (14395-1-AP, 1/1000, Proteintech, Rosemont, IL, USA) was added, followed by overnight incubation in the dark at 4°C. The next day, cells underwent another 1 h incubation at 37°C with the FITC-conjugated secondary antibody (SA00003-8, 1/100, Proteintech). Nuclear staining was achieved with DAPI (Sigma-Aldrich). The slides were observed under an inverted axiovert 200 fluorescence microscope (Carl Zeiss, Gottingen, Germany), and cell surface area was calculated using Image-Pro Plus 6.0 software.

### TOP/FOP flash assay

Topflash or FOPflash luciferase reporters (EMD Millipore, Billerica, MA, USA) were used to detect the activity of the Wnt signaling pathway. In brief, after indicated treatment or transfection, H9c2 cells were plated (5000 cells/well) into 24-well plates and serum-starved overnight. Thereafter, cells were transfected with 0.2 µg TOPflash or FOPflash expression plasmids using Lipofectamine 2000. TOP/FOP luciferase activities were measured using the Luciferase Assay System (Promega, Madison, WI, USA) 48 h after transfection.

### Statistical analysis

All data from at least three repeated individual experiments were analyzed using GraphPad Prism version 5.0 software (GraphPad Software Inc., La Jolla, CA, USA) and are presented as the mean ± standard deviation (SD). Two and multiple comparisons were performed with unpaired Student’s *t-*test and one-way ANOVA followed by the *post hoc* Tukey test. A value of p<0.05 was considered statistically significant.

## Results

### The mortality rate of mice

All animals tolerated the surgery, and no mice died during the operation. After three weeks of the TAC surgery and AAV injection, 1 mouse, 12 mice, 11 mice, and 6 mice died in the Sham group, the TAC group, the TAC + AAV9-shCCAT2 group, and the TAC + AAV9-shNC group, with the survival rate being 90%, 40%, 45%, and 70% respectively.

### CCAT2 is upregulated in the cardiac tissues of CH mouse models and Ang II-induced hypertrophic H9c2 cells

The hearts of mice were harvested for histological examination at 4 weeks post-TAC operation. As shown by the images of H&E staining, TAC surgery markedly enlarged the cross-sectional area of cardiomyocytes in mice (Figure 2A). The echocardiographic measurement reflected that LVEDd and LVESd of post-TAC hearts at 3 weeks were significantly higher while LVEF and LVFS were lower than those of sham hearts (Figure 2B-E), indicating the impaired cardiac function of TAC-operated mice. TAC triggered an obvious increase in heart weight (HW) to body weight (BW) ratio, left ventricle weight (LVW) to BW ratio, and LVW to tibial length (TL) ratio, showing a notably larger proportion of cardiac tissue in CH mice (Figure 2F-H). Besides, considerably upregulated expression of CH markers (β-MHC, ANP, and BNP) was observed in the cardiac tissues of mice after TAC induction (Figure 2I-K). Afterwards, the expression of LncRNA CCAT2 in mouse cardiac tissues after TAC operation was detected by RT-qPCR, which revealed that CCAT2 was overexpressed in hypertrophic heart tissues versus normal heart tissues (Figure 2L), implying that LncRNA CCAT2 might participate in CH development. To further confirm the function of CCAT2 in CH, a cellular model was constructed by treating rat myocardial cells H9c2 with Ang II. The size of H9c2 cells was evaluated through α-SMA immunofluorescence staining, which manifested that Ang II treatment demonstrably cell area of rat cardiomyocytes (Figure 2M). Meanwhile, Ang II-induced hypertrophic cardiomyocytes exhibited significantly higher ANP, BNP, and β-MHC mRNA expression than control cardiomyocytes (Figure 2N). Importantly, LncRNA CCAT2 expression in H9c2 cells was discovered to be remarkably increased upon Ang II treatment (Figure 2O), which further implies the potential role of CCAT2 in CH development.

### Silencing of CCAT2 alleviates CH and cardiac fibrosis in vivo

Next, to determine the detailed role of CCAT2 in regulating CH *in vivo*, AAV9-shCCAT2 was injected into TAC-induced CH model mice through the tail vein to knock down CCAT2 (Figure 3A). After three weeks, we detected and found that the TAC-induced increase in CCAT2 expression in mouse heart tissues was reversed after CCAT2 depletion (Figure 3B). H&E staining illustrated that injection with AAV9-shCCAT2 reduced cardiomyocyte surface area in TAC-induced CH mice (Figure 3C). As an important feature of pathological CH, cardiac fibrosis was detected through Masson’s trichrome staining. Compared with sham mice, mice subjected to TAC surgery presented interstitial and perivascular fibrotic changes in cardiac tissues, accompanied by notable collagen accumulation. However, the above TAC-induced cardiac fibrotic changes were ameliorated after CCAT2 knockdown (Figure 3D). The echocardiographic analysis showed that TAC-induced elevation in LVEDd and LVESd and reduction in LVEF and LVFS of mouse hearts were markedly reversed after downregulating CCAT2 (Figure 3E-H). Moreover, the elevation in HW/BW, LVW/BW, and LVW/TL ratios in mice caused by TAC operation was overturned by silencing of CCAT2 (Figure 3I). Western blotting demonstrated that the TAC-induced increment in β-MHC, ANP, and BNP protein levels in mouse cardiac tissues was antagonized by injection with AAV9-shCCAT2 (Figure 3J-L). Overall, CCAT2 silencing effectively mitigates CH in mice.

**Figure 3:** – Silencing of CCAT2 alleviates CH in model mice. (A) Protocol for AAV9-shCCAT2 or AAV9-shNC injection into TAC-induced CH model mice. (B) Detection of CCAT2 expression in cardiac tissues of CCAT2-silencing mice 3 weeks after TAC surgery by RT-qPCR. n=6 mice per group. (C) Representative H&E staining images of heart sections and quantification of cardiomyocyte cross-sectional area in sham mice and TAC-induced CH mice with or without CCAT2 knockdown. Scale bar, 20 μm. n=6 mice per group. (D) Representative Masson’s trichrome staining images of heart sections and quantification of myocardial fibrosis. Scale bar, 50 μm. n=6 mice per group. (E-H) The LVEDd, LVES, LVEF, and LVFS of mouse hearts. n=6 mice per group. (I) The HW/BW, LVW/BW, and LVW/TL ratio of mice. n=6 mice per group. (J-L) Determination of β-MHC, ANP, and BNP protein levels in mouse cardiac tissues through western blotting. n=6 mice per group. Data are expressed as the means ± SD. *P < 0.05, **p < 0.01, ***p < 0.001 vs Sham; #p < 0.05, ###p < 0.001 vs TAC.

### Silencing of CCAT2 ameliorates cardiomyocyte hypertrophy in vitro

Similarly, the impacts of CCAT2 knockdown on cardiomyocyte hypertrophy *in vitro* were further evaluated after Ang II-treated H9c2 cells were transfected with shCCAT2. We discovered from RT-qPCR that shCCAT2 transfection debilitated the promotion of Ang II treatment on CCAT2 level in H9c2 cells (Figure 4A). As revealed by α-SMA immunofluorescence staining, the knockdown of CCAT2 obviously reduced the surface area in hypertrophic H9c2 cells (Figure 4B-C). Additionally, CCAT2 deficiency counteracted the enhancement in β-MHC, ANP, and BNP levels caused by Ang II treatment in H9c2 cells (Figure 4D-G). These data suggest that CCAT2 silencing improves cardiomyocyte hypertrophy *in vitro*.

**Figure 4:** – Silencing of CCAT2 ameliorates cardiomyocyte hypertrophy in vitro. Ang II-stimulated H9c2 cells were transfected with shCCAT2 or shNC. (A) Analysis of CCAT2 expression in H9c2 cells through RT-qPCR. n=3 repetitions. (B-C) Assessment of the size of H9c2 cells by α-SMA immunofluorescence staining. Scale bar, 20 μm. n=3 repetitions. (D-G) Examination of β-MHC, ANP, and BNP levels in H9c2 cells by western blotting and RT-qPCR. n=3 repetitions. Data are expressed as the means ± SD. ***P < 0.001 vs Control; #p < 0.05, ##p < 0.01, ###p < 0.001 vs Ang II.

### Silencing of CCAT2 inhibits the activation of the Wnt/β-catenin signaling in CH

To Figure out the molecular mechanism through which CCAT2 knockdown plays a protective role against CH, the influence of CCAT2 depletion on the Wnt/β-catenin signaling was estimated in both CH mouse models and hypertrophic cardiomyocytes. As shown in Figure 5A-B, TAC surgery resulted in the Wnt/β-catenin pathway activation in CH mice, which however, was suppressed by depletion of CCAT2, as evidenced by the reduction in active β-catenin and phosphorylated-GSK-3β protein levels in CCAT2-silenced CH mice. The expression of Wnt target genes was detected through RT-qPCR, which depicted that c-Myc, cyclinD1, and c-Jun expression in mouse cardiac tissues was markedly enhanced after TAC operation, but was attenuated after AAV9-shCCAT2 injection (Figure 5C). Furthermore, the inactivation of CCAT2 knockdown on the Wnt/β-catenin pathway in CH was validated *in vitro*. Ang II-induced increment in active β-catenin and phosphorylated-GSK-3β levels in H9c2 cells was offset by the downregulation of CCAT2 (Figure 5D-E). RT-qPCR illustrated that Ang II-induced upregulation in c-Myc, cyclinD1, and c-Jun expression in H9c2 cells was reversed by CCAT2 knockdown (Figure 5F). More importantly, we observed a significant decline in TOP/FOP ratio in hypertrophic H9c2 cells, while CCAT2 silencing pronouncedly abrogated this effect caused by Ang II (Figure 5G). Jointly, CCAT2 knockdown plays an inhibitory role on the Wnt/β-catenin pathway in CH.

**Figure 5:** – Silencing of CCAT2 inhibits the activation of the Wnt/β-catenin pathway in CH. (A-B) Measurement of active β-catenin and phosphorylated-GSK-3β protein levels in sham mice and TAC-induced CH mice with or without AAV9-shCCAT2 or AAV9-shNC injection by western blotting. n=6 mice per group. (C) Detection of c-Myc, cyclinD1, and c-Jun mRNA levels in TAC-induced CH mice after silencing of CCAT2 by RT-qPCR. n=6 mice per group. Data are expressed as the means ± SD. **P < 0.01, ***p < 0.001 vs Sham; #p < 0.05, ###p < 0.001 vs TAC. (D-E) Examination of active β-catenin and phosphorylated-GSK-3β protein levels in control H9c2 cells and Ang II-stimulated H9c2 cells with or without shCCAT2 or shNC transfection via western blotting. n=3 repetitions. (F) Assessment of c-Myc, cyclinD1, and c-Jun mRNA levels in Ang II-treated H9c2 cells after knockdown of CCAT2 by RT-qPCR. n=3 repetitions. (G) Evaluation of the Wnt/β-catenin pathway activity in H9c2 cells following Ang II treatment and shCCAT2 or shNC transfection by TOP/FOP flash assay. n=3 repetitions. Data are expressed as the means ± SD. ***P < 0.001 vs Control; #p < 0.05, ###p < 0.001 vs Ang II.

### Silencing of CCAT2 relieves cardiomyocyte hypertrophy in vitro by restraining the Wnt/β-catenin pathway

To validate whether CCAT2-mediated cardiomyocyte hypertrophy is dependent on the Wnt/β-catenin pathway, H9c2 cells were pretreated with LiCl, an agonist of the Wnt/β-catenin pathway, before Ang II treatment. According to α-SMA immunofluorescence staining, we found that the inhibition of CCAT2 silencing on the surface area of hypertrophic H9c2 cells was overturned by LiCl pretreatment (Figure 6A-B). As illustrated by western blotting, pretreatment with LiCl reversed the reduction in β-MHC, ANP, and BNP protein levels caused by CCAT2 depletion in hypertrophic H9c2 cells (Figure 6C-E). The results of RT-qPCR were in line with those of western blotting, which demonstrated that LiCl abolished the shCCAT2-induced decline in β-MHC, ANP, and BNP mRNA levels in hypertrophic H9c2 cells (Figure 6F). Overall, LiCl blunts the preventive effects of CCAT2 silencing on Ang II-induced cardiomyocyte hypertrophy.

**Figure 6:** – Silencing of CCAT2 relieves cardiomyocyte hypertrophy in vitro by restraining the Wnt/β-catenin pathway. H9c2 cells were pretreated with LiCl before Ang II stimulation and shCCAT2 or shNC transfection. (A-B) Determination of H9c2 cell size through α-SMA immunofluorescence staining. n=3 repetitions. Scale bar, 20 μm. (C-F) Assessment of β-MHC, ANP, and BNP levels in H9c2 cells through western blotting and RT-qPCR. n=3 repetitions. (G) Schematic diagram illustrating the underlying molecular mechanism of LncRNA CCAT2 in CH. Data are expressed as the means ± SD. ***P < 0.001 vs Control; #p < 0.05, ###p < 0.001 vs Ang II; &&p < 0.01, &&&p < 0.001 vs Ang II + shCCAT2.

## Discussion

This study provides evidence supporting the alleviative effects of LncRNA CCAT2 downregulation on CH. Silencing of CCAT2 has been demonstrated to blunt Ang II-induced cardiomyocyte hypertrophy *in vitro* and ameliorate pressure overload-induced myocardial hypertrophy in mouse models. Mechanically, CCAT2 knockdown inactivated the Wnt/β-catenin signaling, and supplementation of LiCl, an agonist of the Wnt/β-catenin signaling, abated the anti-hypertrophic effect of CCAT2 silencing. The above evidence indicates that silencing of CCAT2 exerts an anti-hypertrophic effect in CH, which relies on its interruption of the Wnt/β-catenin signaling.

Accumulating evidence has proved that LncRNAs exert either promotive or inhibitory effects on the progression of CH through a diversity of mechanisms. For example, Wen et al. suggested that PEG10 was upregulated in pathological CH, and PEG10 aggravated CH through increasing HOXA9 expression.^21^ Zhou et al. discovered that UCA1 was highly expressed in the constructed hypertrophic cardiomyocytes and CH murine models, and UCA1 facilitated CH progression by enhancing HOXA9 expression through competitively binding with miR-184.^22^ In contrast, other studies have indicated the protective effects of some LncRNAs in the development of CH. Song et al. disclosed that ADAMTS9-AS1 expression was reduced in hypertrophic obstructive cardiomyopathy patients and isoproterenol-treated human cardiomyocyte cells, and overexpression ADAMTS9-AS1 repressed cardiomyocyte hypertrophy by upregulating KAT7 through sponging miR-185-5p.^23^ Wang et al. reported that both cellular and animal models of CH presented decreased expression of H19, and H19 overexpression hindered CH development via modulating the miR-145-3p/SMAD4 axis.^24^ Previously, the detailed role and molecular mechanism of LncRNA CCAT2 in human diseases, especially cancers, have been elucidated. For example, deficiency of CCAT2 restrains cell growth and metastasis but induces G0/G1 arrest and apoptosis in breast cancer through inactivating the TGF-β signaling pathway.^25^ CCAT2 attenuates radiosensitivity and apoptosis of esophageal cancer cells by negatively regulating the miR-145/p70S6K1 axis and the p53 signaling pathway.^26^ CCAT2 promotes gastric cancer cell growth and metastasis through modulating CD44 alternative splicing via binding to ESRP1.^27^ Nonetheless, whether CCAT2 exerts regulatory effects in CH has not been explored before. Herein, we discovered that LncRNA CCAT2 level was considerably higher in TAC-induced CH mouse models and Ang II-treated H9c2 cells. Furthermore, silencing of CCAT2 substantially alleviated CH in mice and hypertrophic H9c2 cells, which was validated by its inhibition of the expression of key CH markers (β-MHC, ANP, and BNP). This suggests the enhancing effect of LncRNA CCAT2 on the progression of CH.

The canonical Wnt/β-catenin signaling pathway is well known for its regulatory role in embryonic development, stem cell homeostasis, and tissue regeneration.^28^ In recent years, accumulating evidence has demonstrated that the abnormal activation of this pathway in the heart underlies the pathophysiology of myocardial hypertrophy and myocardial injury.^29^ It has been confirmed that β-catenin participates in the metabolism of cardiomyocytes, thereby affecting the function and development of the heart.^30^ Studies have proved that β-catenin overexpression can result in CH.^31,32^ Overexpressing β-catenin in cardiac myocytes increases cell volume, induces actin formation, activates pathological hypertrophy markers, and elevates ANP and BNP levels.^33^ CH can be ameliorated through knockout or inhibition of myocardial β-catenin.^34^ Zhao et al. reported that multiple Wnt ligands were induced and β-catenin was activated in rats with chronic infusion of Ang II and rat primary cardiomyocytes, and blockade of the Wnt/β-catenin pathway by ICG-001 attenuated CH and myocardial fibrosis as well as inhibited hypertrophic marker gene expression.^35^ Therefore, suppression of the Wnt/β-catenin signaling might be considered a promising therapeutic target for CH. Previously, many studies have unraveled that CCAT2 participates in mediating cancer development via modulating the Wnt/β-catenin signaling. For instance, CCAT2 knockdown restrains cell growth and metastasis and facilitates cell cycle arrest and apoptosis in thyroid cancer by attenuating Wnt/β-catenin cascade activity.^36^ The inhibitory effects on prostate cancer cell growth, cell cycle, epithelial-mesenchymal transition, and metastasis induced by silencing of CCAT2 were antagonized by treatment with the Wnt/β-catenin pathway activator LiCl.^37^ Downregulation of CCAT2 impedes oral squamous cell carcinoma cell malignant biological behaviors via inactivating the Wnt/β-catenin signaling.^38^ Based on the above literature, we speculated that CCAT2 might also mediate CH development by modulating the Wnt/β-catenin signaling. Herein, we observed that the upregulation in the levels of active β-catenin and phosphorylated-GSK-3β and Wnt target genes c-Myc, cyclinD1, and c-Jun induced by TAC in CH mice and by Ang II in hypertrophic cardiomyocytes was overturned by silencing of CCAT2. Importantly, LiCl treatment abrogated the suppression of CCAT2 knockdown on cell surface area and β-MHC, ANP, and BNP levels in hypertrophic H9c2 cells, suggesting that CCAT2 silencing hampers CH progression by inactivating the Wnt/β-catenin signaling.

To be honest, there exist some limitations in our study. First, when measuring the size of mouse cardiomyocytes, the heart should be in diastole, so the area of cardiomyocytes we obtained may be smaller than the actual size. Second, to further elucidate the role of CCAT2 in the pathogenesis of CH, different cell type-specific conditional silencing of CCAT2 mice are required for further investigations. Third, the expression of CCAT2 in human heart failure tissues remains uncertain. Tissue specimens from patients with end-stage heart failure should be collected and analyzed in future studies. Finally, further studies are warranted to explore the detailed underlying mechanism through which CCAT2 inactivates the Wnt/β-catenin signaling pathway during CH.

To date, this is the first report to demonstrate that silencing CCAT2 has an inhibitory effect on Ang II-induced cardiomyocyte hypertrophy *in vitro* and TAC-induced CH *in vivo* by repressing the Wnt/β-catenin signaling (Figure 6G). CCAT2 has the potential to provide a target for the development of more effective clinical therapeutics for CH.
